# Syntheses and crystal structures of hydrated and anhydrous 1:2 cocrystals of oxyresveratrol and zwitterionic proline

**DOI:** 10.1107/S2056989020011536

**Published:** 2020-08-28

**Authors:** Passaporn Ouiyangkul, Saowanit Saithong, Vimon Tantishaiyakul

**Affiliations:** a Department of Pharmaceutical Chemistry, Faculty of Pharmaceutical Sciences, Prince of Songkla University, Hat-Yai, 90112, Thailand; b Division of Physical Science and Center of Excellence for Innovation in Chemistry, Faculty of Science, Prince of Songkla University, Hat-Yai, Songkhla, 90112, Thailand; c Medical Science Research and Innovation Institute, Research and Development Office, Prince of Songkla University, Hat-Yai, 90112, Thailand; d Center of Excellence for Drug Delivery System and Department of Pharmaceutical, Chemistry, Faculty of Pharmaceutical Sciences, Prince of Songkla University, Hat-Yai, 90112, Thailand

**Keywords:** cocrystal structure, zwitterion, oxyresveratrol, resveratrol

## Abstract

The hydrated and anhydrous 1:2 cocrystals of oxyresveratrol (4-[(*E*)-2-(3,5-di­hydroxy­phen­yl)ethen­yl]benzene-1,3-diol; OXY; C_14_H_12_O_4_) and proline [(*S*)-pyrrolidine-2-carb­oxy­lic acid; PRO; C_5_H_9_NO_2_], namely, 4-[(*E*)-2-(3,5-di­hydroxy­phen­yl)ethen­yl]benzene-1,3-diol bis­[(*S*)-pyrrolidin-1-ium-2-carboxyl­ate] monohydrate, C_14_H_12_O_4_·2C_5_H_9_NO_2_·H_2_O, and the anhydrous form, C_14_H_12_O_4_·2C_5_H_9_NO_2_, were obtained by crystallization at different temperatures. Both of them crystallize with ortho­rhom­bic (*P*2_1_2_1_2_1_) symmetry. The structures display N—H⋯O and O—H⋯O hydrogen-bonding inter­actions between PRO and PRO, OXY and OXY, and OXY and PRO. In the hydrated cocrystal, these types of contacts are also observed between the OXY, PRO and water mol­ecules. A combination of these inter­actions leads to a three-dimensional supra­molecular assembly in each case. Hirshfeld surfaces were used to gain further insight into the inter­molecular inter­actions in the packing, including the relative percentage contributions of the significant inter­molecular H⋯H and H⋯O/O⋯H contacts.

## Abstract 2

The hydrated and anhydrous 1:2 cocrystals of oxyresveratrol and proline, which were prepared by crystallizations at different temperatures, show similar packing motifs.

## Chemical context   

Oxyresveratrol (4-[(*E*)-2-(3,5-di­hydroxy­phen­yl)ethen­yl]benzene-1,3-diol; OXY; C_14_H_12_O_4_) is a natural stilbenoid found in various plants, such as *Morus alba* L. (Lu *et al.*, 2017 ▸). It has several biological activities, including neuroprotective and hepatoprotective effects (Shah *et al.*, 2019 ▸; Jia *et al.*, 2018 ▸; Chao *et al.*, 2008 ▸). As the aqueous solubility of OXY is low, there have been attempts to improve its solubility and oral bioavailability by cocrystallization with citric acid and glutaric acid (Suzuki *et al.*, 2019 ▸). Proline [(*S*)-pyrrolidine-2-carb­oxy­lic acid; PRO,; C_5_H_9_NO_2_] is a natural amino acid that has a secondary amino group in the form of a pyrrolydinic ring. It is an osmoprotectant and is used frequently in many pharmacological and biotechnological applications (Panday, 2011 ▸). PRO has been used as a cocrystal former in various drugs and pharmacological active compounds because of its mol­ecular rigidity and high solubility in water (Chesna *et al.*, 2017 ▸; Surov *et al.*, 2018 ▸; Tilborg *et al.*, 2014 ▸). According to previous studies, the phenolic hydroxyl groups of flavonoids are able to form charge-assisted hydrogen bonds with the carboxyl­ate moiety of PRO (He *et al.*, 2016 ▸). Moreover, PRO could form a cocrystal with resveratrol [(*E*)-5-(4-hy­droxy­styr­yl)benzene-1,3-diol; RES; C_14_H_12_O_3_], which is a close analogue of OXY (He *et al.*, 2017 ▸). Therefore, PRO is a good candidate as a cocrystal former for cocrystallization with OXY and we now describe the syntheses and structures of hydrated and anhydrous 1:2 cocrystals of OXY and PRO, hereafter (I) and (II).
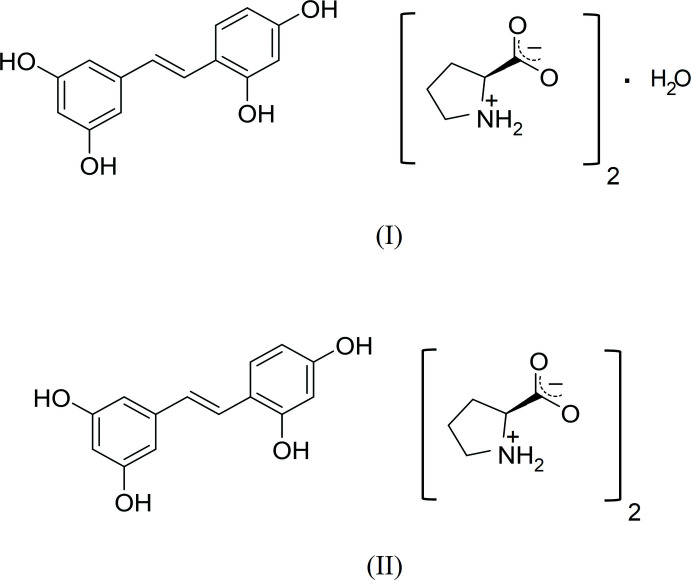


## Structural commentary   

**Figure 1:**
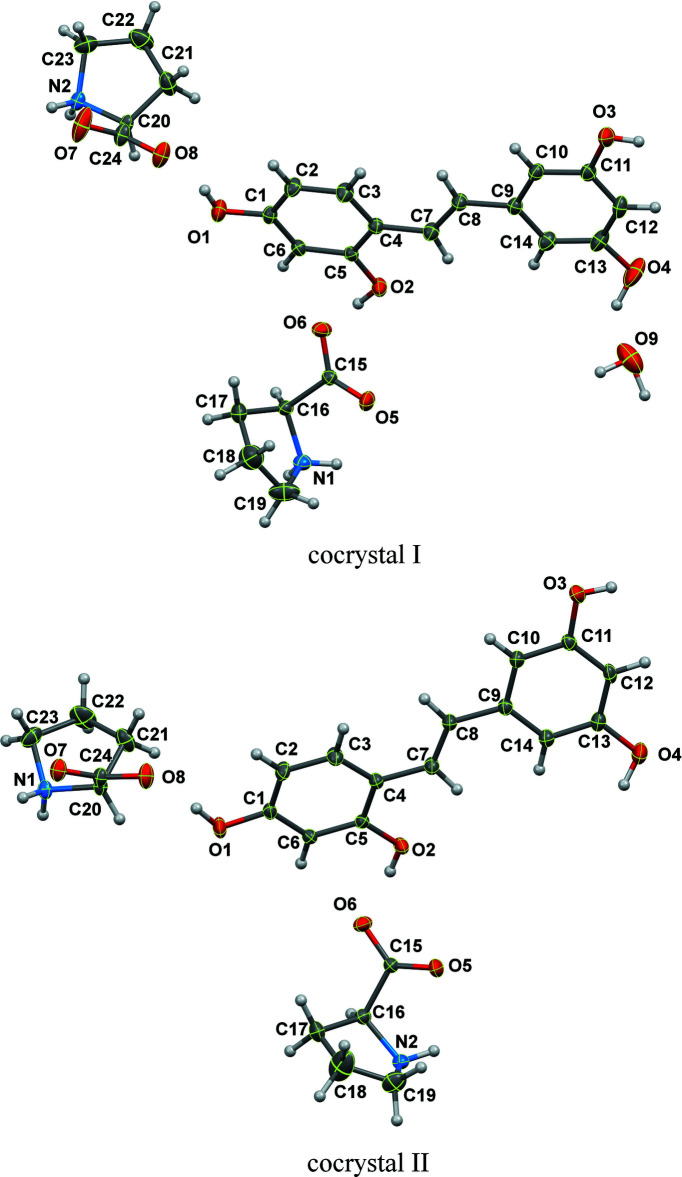
The mol­ecular structures of (I) and (II) showing 50% displacement ellipsoids.

Both cocrystals of OXY and PRO form a 1:2 stoichiometry in the ortho­rhom­bic system, space group *P*2_1_2_1_2_1_, with *Z* = 4. The asymmetric unit of (I) contains two PRO, one OXY and one water mol­ecule while the asymmetric unit of (II) consists of only two PRO and one OXY mol­ecules, as depicted in Fig. 1 ▸. The dihedral angle between the planes of the OXY C1–C6 and C9–C14 phenyl rings in (I) is 7.1 (2)°. This is slightly different from the previous report [9.39 (9)°] of the corresponding angle in OXY·2H_2_O (Deng *et al.*, 2012 ▸). However, a more twisted dihedral angle between these phenyl rings is observed in (II), of 14.15 (19)°. It might be caused by the influence of hydrogen-bonding inter­actions in the crystal. In addition, the zwitterionic form of the PRO mol­ecules of both cocrystals is confirmed by the C—O and C—N bond lengths.

## Supra­molecular features   

**Figure 2:**
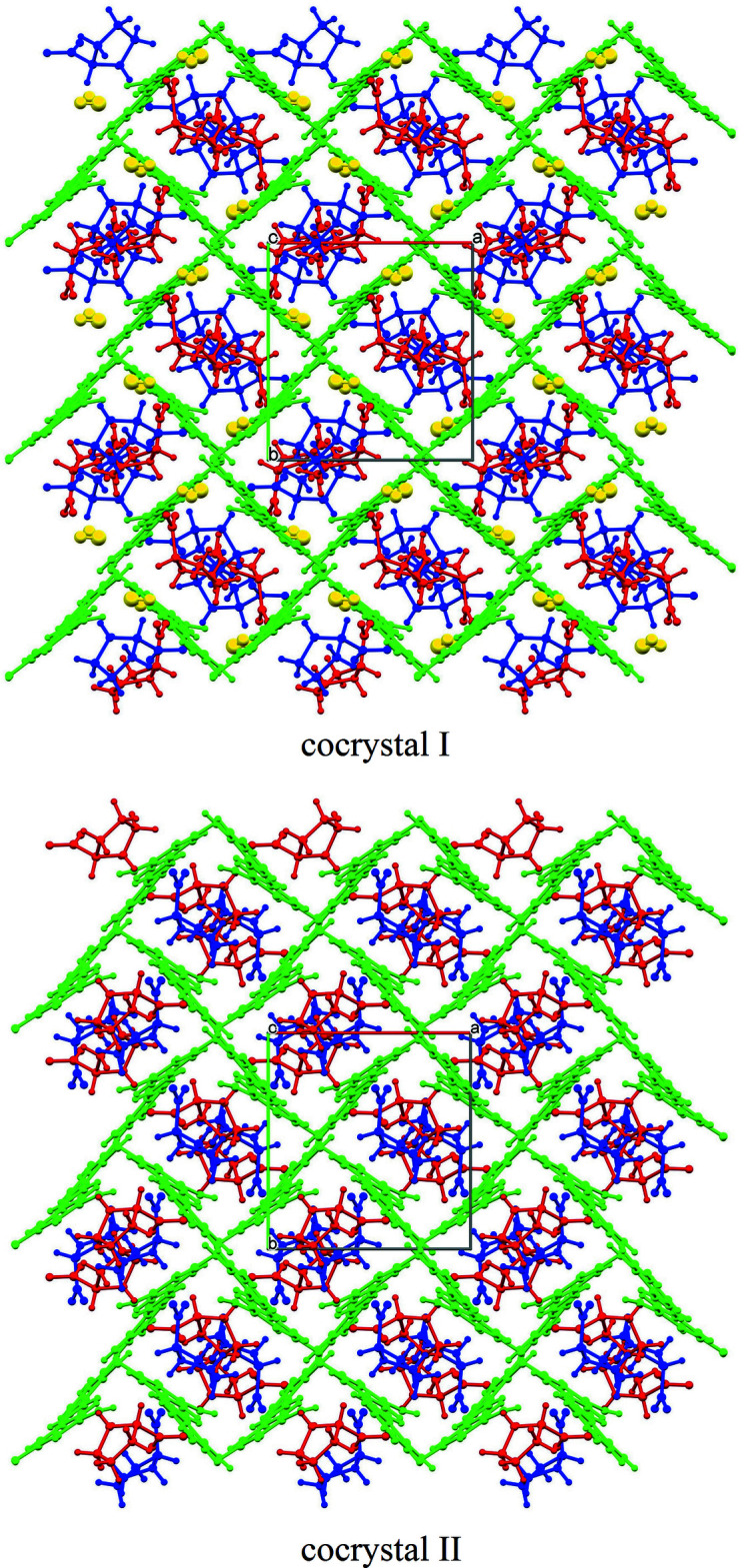
The packing in (I) and (II); colour code PRO 1 (blue), PRO 2 (red), OXY (green) and water (yellow).

The packing for (I) and (II) is shown in Fig. 2 ▸. The two PRO mol­ecules (PRO 1 and PRO 2) are indicated in blue and red, respectively, whereas the OXY and water mol­ecules are shown in green and yellow, respectively. The main architectures of (I) and (II) are quite similar but there are clearly differences regarding the water mol­ecule in (I).

**Figure 3:**
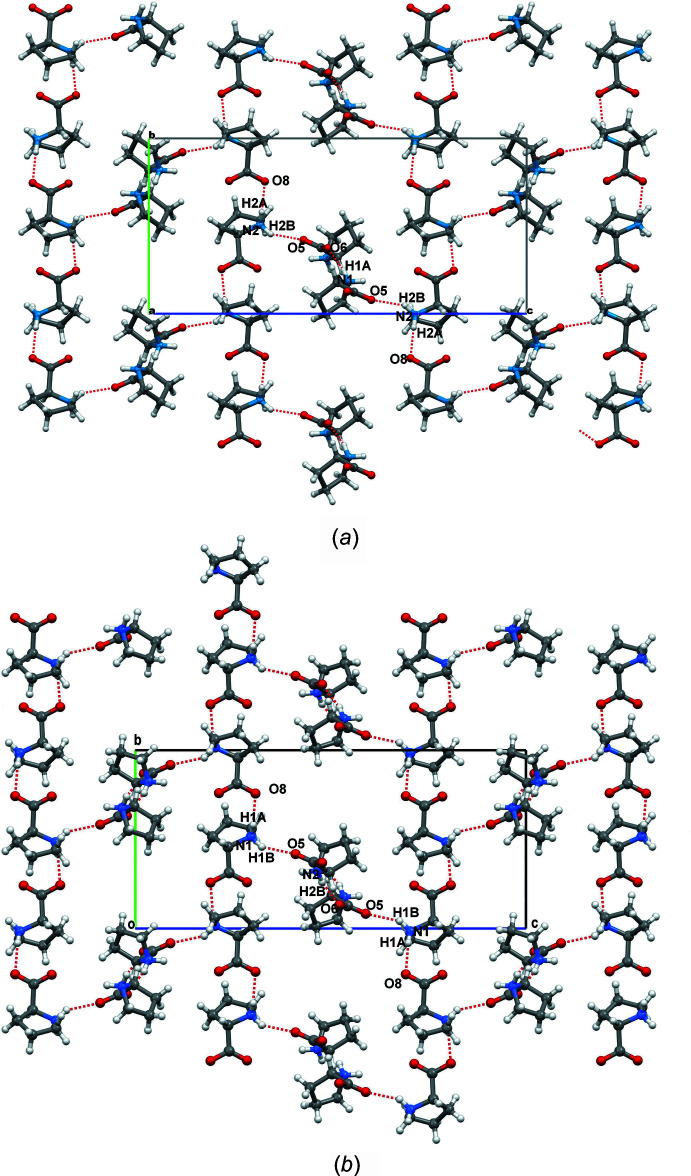
Hydrogen-bond inter­actions between the PRO mol­ecules viewed down [100] in (*a*) (I) and (*b*) (II).

The PRO 1 and PRO 2 mol­ecules form a three-dimensional network of N—H⋯O hydrogen bonds between the H atoms of NH_2_ groups and O atoms of carboxyl­ate groups: N1—H1*A*⋯O6^iii^, N2—H2*A*⋯O8^v^ and N2—H2*B*⋯O5^vi^ for (I) and N1—H1*B*⋯O5^iii^, N1—H1*A*⋯O8^iv^ and N2—H2*B*⋯O6^vi^ for (II) (see Tables 1 ▸ and 2 ▸, where the symmetry codes are defined). The hydrogen-bonding inter­actions between PRO 1 and PRO 2 of both cocrystals viewed down [100] are shown in Fig. 3 ▸. The phenolic hydroxyl groups from the OXY mol­ecule inter­act with O atoms of the carboxyl­ate groups of the PRO mol­ecules *via* O—H⋯O hydrogen bonds, namely O2—H2′⋯O6 and O1—H1′⋯O8 for (I) and O1—H1′⋯O8, O2—H2′⋯O6 and O4—H4′⋯O7^ii^ for (II). In addition, one of the four hydroxyl groups of OXY accepts a hydrogen bond N1—H1*B*⋯O4^iv^ from PRO at an N⋯O distance of 2.951 (4) Å in (I) while the equivalent bond in (II) is observed at 2.920 (4) Å for N2—H2*A*⋯O4^v^. Moreover, hydrogen-bonding contacts among the OXY mol­ecules in both cocrystals are observed between phenolic hydroxyl groups, O3—H3′⋯O2^i^, for both cocrystals. Further hydrogen-bond inter­actions involving the water mol­ecule are observed in (I): N1—H1*B*⋯O9^iv^ between PRO and water [N⋯O = 3.105 (5) Å] and O4—H4′⋯O9 [2.575 (6) Å] and O9—H9*A*⋯O7^ii^ [2.639 (5) Å] inter­actions between OXY and water mol­ecules. Taken together, the hydrogen bonds in both cocrystals form complex three-dimensional supra­molecular architectures.

## Hirshfeld surface analysis   

Hirshfeld surface analysis and two dimensional fingerprint plots are used to provide the additional insight of the weak inter­molecular contacts and inter­molecular inter­actions in the crystal packing of mol­ecules (McKinnon *et al.*, 2004 ▸; 2007 ▸; Spackman & Jayatilaka, 2009 ▸). The blue, white and red areas in the *d*_norm_-mapped Hirshfeld surfaces indicate inter­atomic contacts longer, equal to and shorter than the sum of the van der Waals radii, respectively. Analysis of (I) and (II) was performed by using *Crystal Explorer 17.5* (Turner *et al.*, 2017 ▸). The Hirshfeld surfaces are plotted for individual components, to examine the inter­actions of the main mol­ecules (PRO and OXY) in the cocrystals.

**Figure 4:**
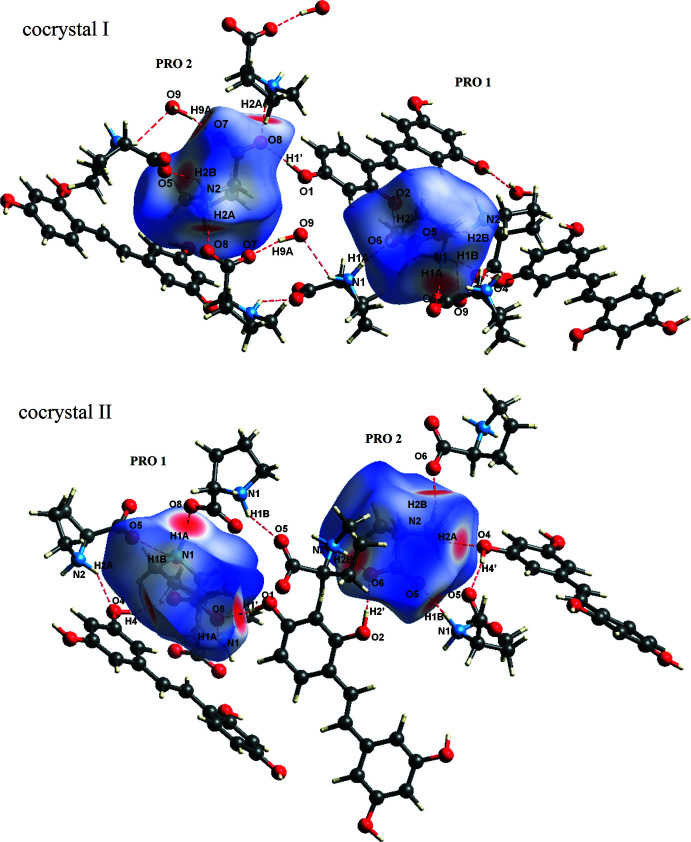
Hirshfeld surfaces of the PRO mol­ecules in (I) and (II).

The Hirshfeld surfaces around the PRO mol­ecules mapped over *d*_norm_ are shown in Fig. 4 ▸ with selected atoms labelled (compare Tables 1 ▸ and 2 ▸). There are red spots on the surface close to H atoms of the amine group inside the surface of PRO mol­ecules in both cocrystals, H1*A* and H1*B* for PRO 1 and H2*A* and H2*B* for PRO 2. The inside zones indicate hydrogen-bond donors to acceptor O atoms at outside surfaces of the nearby carboxyl­ate groups of adjacent PRO mol­ecules (N—H⋯O type), hydroxyl group of OXY (N—H⋯O type) and O atoms of water [only for (I), O—H⋯O and N—H⋯O forms]. Besides, the O atoms of the carboxyl­ate groups of both PRO mol­ecules acting as hydrogen-bond acceptors inter­act with the hydrogen-bond donor NH_2_ group of PRO mol­ecules on the outside surfaces, as discussed in the *Supra­molecular features* section.

**Figure 5:**
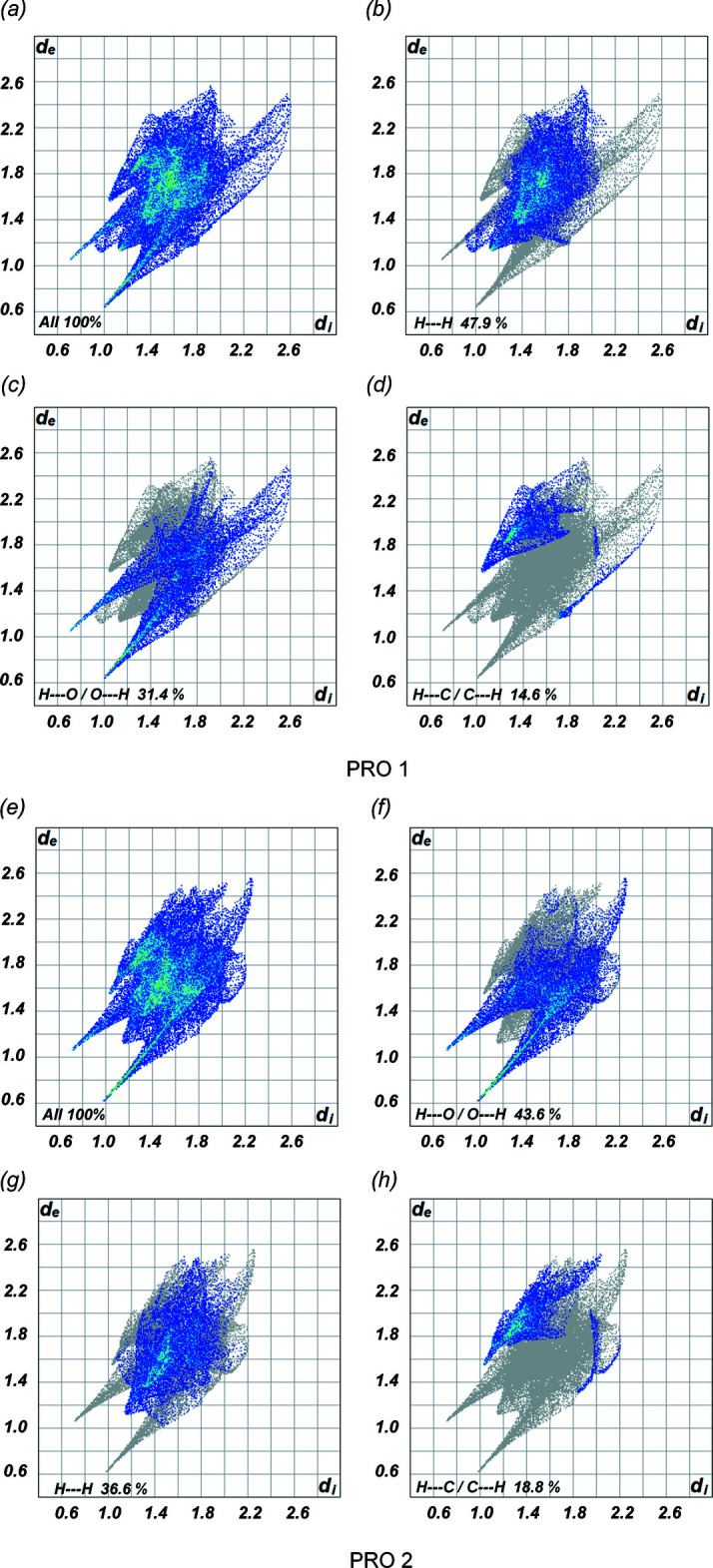
Fingerprint plots for the PRO mol­ecules in (I): (*a*)–(*d*) of PRO 1 and (*e*)–(*h*) of PRO 2.

In addition, the two-dimensional fingerprint plots of the PRO mol­ecules for (I) and (II) are illustrated in Figs. 5 ▸ and 6 ▸, showing the relative contributions of the various types of contacts to the Hirshfeld surface. The overall fingerprint plot for PRO 1 is shown in Fig. 5 ▸ *a* and 6*a* and those delineated into the contacts of H⋯H, O⋯H/H⋯O and C⋯H/H⋯C inter­actions are displayed in Fig. 5 ▸ *b*–*d* and 6*b*–*d*. Similarly, the overall fingerprint plot of PRO 2 of both cocrystals is presented in Fig. 5 ▸ *e* and 6*e* and those delineated into individual contacts are shown in Fig. 5 ▸ *f*–*h* and 6*f*–*h*. For cocrystals (I) and (II), the most significant inter­actions in terms to their relative percentage contributions are by H⋯H contacts with the second largest percentage attributed to H⋯O/O⋯H inter­actions in one PRO mol­ecule and *vice versa* for the other PRO mol­ecule. A pair of blue-colored spikes pointing towards the bottom left of the H⋯O/O⋯H contacts in Figs. 5 ▸ and 6 ▸ correlate with the important O—H⋯O and N—H⋯O hydrogen bonds associated with the deep-red spots shown in Fig. 4 ▸. The asymmetric pair of wings for H⋯C/C⋯H inter­actions in both cocrystals are also found, while other types of contact make a negligible contribution. The relative percentage contributions for the PRO 1 and PRO 2 mol­ecules in both cocrystals are summarized in Table 3 ▸.

**Figure 7:**
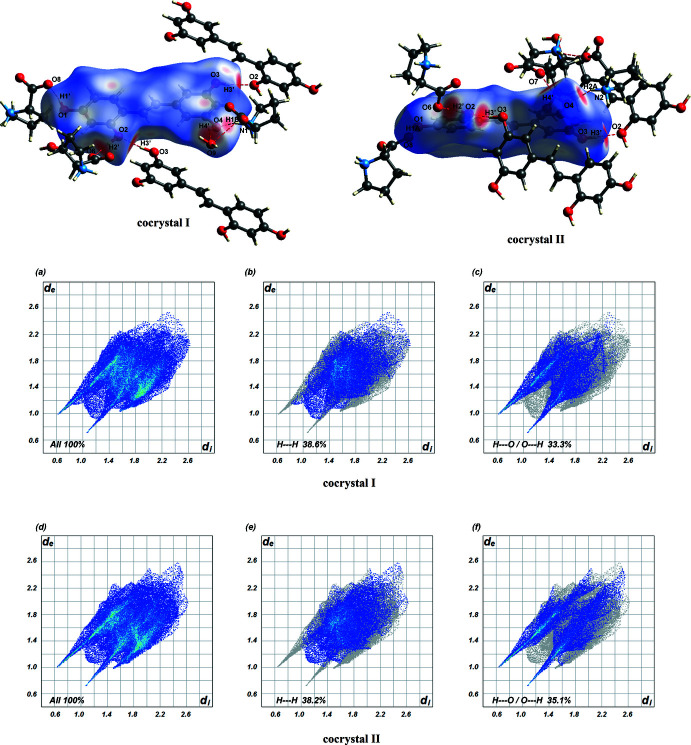
Hirshfeld surfaces and fingerprint plots for the OXY mol­ecules; (*a*)–(*c*) refer to (I) and (*d*)–(*f*) refer to (II).

The OXY Hirshfeld surface, including fingerprint plots for each cocrystal, is depicted in Fig. 7 ▸. The bright-red spots on the surfaces relate to the significant hydrogen bonds of the phenolic hydroxyl groups as O donors (O—H⋯O) and acceptors (N—H⋯O). In (I), the hydrogen-bond contacts are observed from the O atom of the water mol­ecule linking with the OXY surface through one of the hydroxyl groups. In addition, it is found that this water mol­ecule is connected with PRO mol­ecule *via* a hydrogen-bonding inter­action, as indicated in part of the PRO surfaces. The fingerprint plots for OXY are illustrated below the Hirshfeld surfaces in Fig. 7 ▸ *a*–*c* for (I) and Fig. 7 ▸ *d*–*f* for (II). The fingerprint plots Fig. 7 ▸ *a* and Fig. 7 ▸ *d* show the overall inter­actions (100%) of the OXY surface. The most significant inter­actions are H⋯H contacts [38.6% for (I) and 38.2% for (II)] and the second largest percentage [33.3% for (I) and 35.1% for (II)] can be attributed to H⋯O/O⋯H contacts, which are seen as red spots on the Hirshfeld surfaces and correlate with the O—H⋯O and N—H⋯O hydrogen bonds. The relative percentage contributions of OXY are also included in Table 3 ▸. Overall, there are few differences between the Hirshfeld surfaces, fingerprint patterns and the relative percentage contributions for (I) and (II).

## Database survey   

Based on the SciFinder (2020 ▸) database, there are no reports for cocrystal structures containing OXY. Only the crystal structure of OXY dihydrate was previously reported (Deng *et al.*, 2012 ▸; Cambridge Structural Database refcode ZAPDOL). The connecting C=C bond of OXY has a *trans* configuration and allows the setup of a conjugated system throughout the OXY mol­ecule. Furthermore, in the crystal, the OXY mol­ecules are connected through O—H⋯O hydrogen bonds between the hy­droxy groups of OXY and water mol­ecules. The anhydrous and monohydrate crystals of PRO have been reported in numerous papers (Seijas *et al.*, 2010 ▸; Janczak & Luger, 1997 ▸; Verbist *et al.*, 1972 ▸; Caetano *et al.*, 2018 ▸; Koenig *et al.*, 2018 ▸) and PRO invariably crystallizes in the zwitterionic form.

A search for cocrystal structures of PRO gave 148 hits. PRO has been used as a cocrystal former of various active pharmaceutical ingredients (Tilborg *et al.*, 2013 ▸; Tumanova *et al.*, 2018 ▸; Song *et al.*, 2019 ▸). The most relevant cocrystal structure to this work is the cocrystal of RES and PRO (He *et al.*, 2017 ▸; refcode PEBZEE). RES and PRO form O—H⋯O hydrogen bonds in the cocrystal.

## Synthesis and crystallization   

OXY and PRO were purchased from Chengdu Biopurify Phytochemicals Ltd. (Sichuan, China) and Sigma Aldrich (St. Louis, MO, USA), respectively. All organic solvents used were of analytical grade and were purchased from RCI Labscan Ltd (Bangkok, Thailand). All chemicals and solvents were used as received without further purification. Solid OXY (122.10 mg, 0.50 mmol) and PRO (115.10 mg, 1.00 mmol) were added to a 20 ml transparent glass vial. To this was added a mixture of methanol and aceto­nitrile (1:1 *v*/*v*, 12 ml), followed by sonication until all solids were entirely dissolved. The mixture was divided into two portions, and each was covered with aluminum foil with a few small holes in it. Crystals of (I) in the form of colourless rods were obtained when the solution was placed on a hot plate at 323 K for 16 h. Single crystals of (II) (colourless blocks) were grown from a solution that was left at room temperature (303 K) for 16 h.

## Refinement   

Crystal data, data collection and structure refinement details for (I) and (II) are summarized in Table 4 ▸. The H atoms of PRO mol­ecules of both cocrystals were included with calculated positions and isotropically refined with *U*_iso_(H) = 1.2*U*_eq_(N). However, two H atoms on phenolic hydroxyl groups for OXY [for (I) and (II)] and water [for (I)] were located in difference maps and isotropically refined with the distance restraint O—H = 0.82 (2)–0.89 (2) Å for OXY and O—H = 0.89 (2)–1.03 (2) Å for water. The other two H atoms of the OXY mol­ecules were calculated and isotropically refined and the constraint with *U*_iso_(H) = 1.5*U*_eq_(O) was applied.

## Supplementary Material

Crystal structure: contains datablock(s) I, II, global. DOI: 10.1107/S2056989020011536/hb7935sup1.cif

Structure factors: contains datablock(s) I. DOI: 10.1107/S2056989020011536/hb7935Isup2.hkl

Structure factors: contains datablock(s) II. DOI: 10.1107/S2056989020011536/hb7935IIsup3.hkl

Supporting information file. DOI: 10.1107/S2056989020011536/hb7935Isup4.cml

Supporting information file. DOI: 10.1107/S2056989020011536/hb7935IIsup5.cml

CCDC references: 2024883, 2024882

Additional supporting information:  crystallographic information; 3D view; checkCIF report

## Figures and Tables

**Figure 6:**
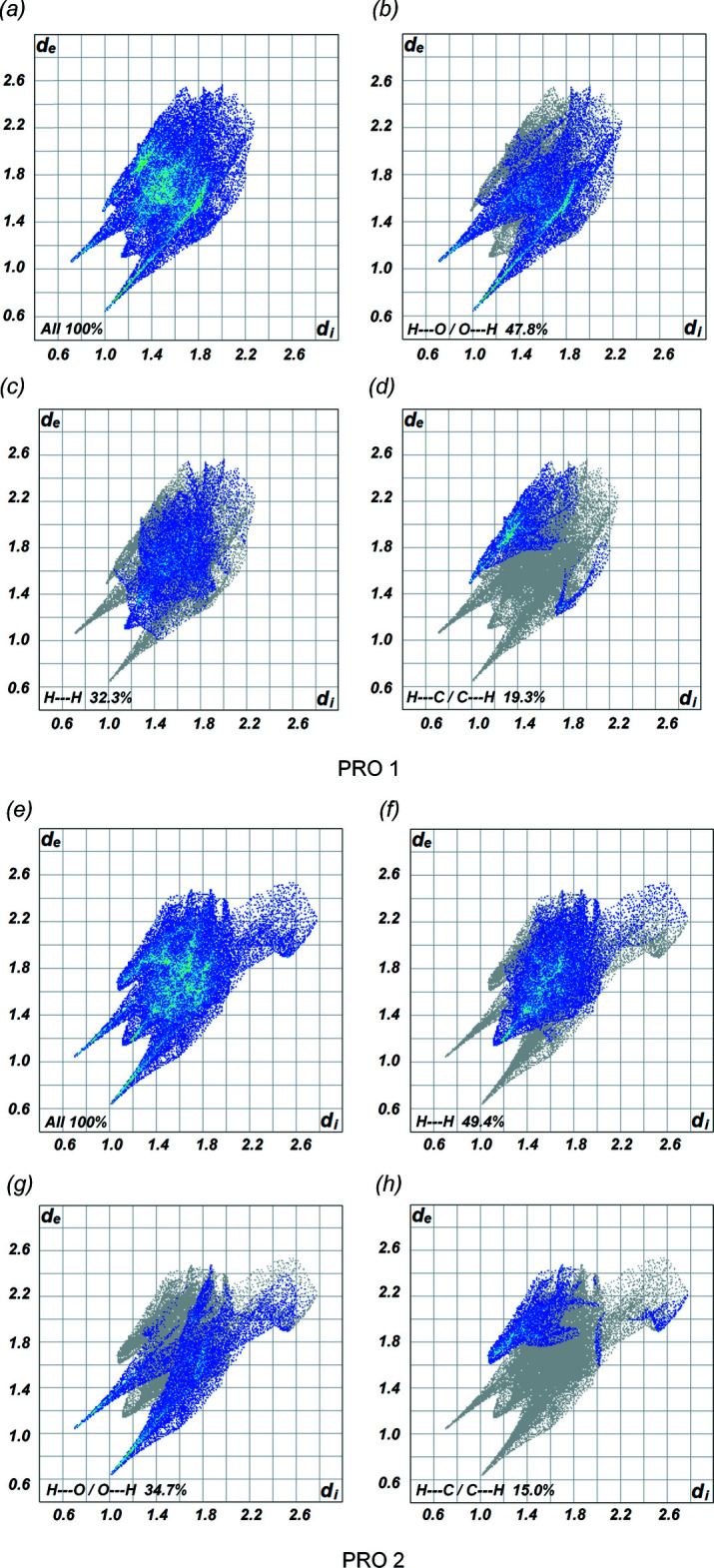
Fingerprint plots for the PRO mol­ecules in (II): (*a*)–(*d*) of PRO 1 and (*e*)–(*h*) of PRO 2.

**Table 1:** Hydrogen-bond geometry (Å, °) for (I)

*D*—H⋯*A*	*D*—H	H⋯*A*	*D*⋯*A*	*D*—H⋯*A*
O1—H1′⋯O8	0.84 (2)	1.78 (3)	2.597 (3)	166 (5)
O2—H2′⋯O6	0.92	1.74	2.647 (3)	172
O3—H3′⋯O2^i^	0.85 (2)	1.93 (3)	2.778 (3)	175 (5)
O4—H4′⋯O9	0.99	1.76	2.575 (6)	137
O9—H9*A*⋯O7^ii^	0.89 (2)	1.76 (3)	2.639 (5)	168 (4)
N1—H1*A*⋯O6^iii^	0.89	1.90	2.777 (4)	168
N1—H1*B*⋯O4^iv^	0.89	2.28	2.951 (4)	132
N1—H1*B*⋯O9^iv^	0.89	2.47	3.105 (5)	129
N2—H2*A*⋯O8^v^	0.89	1.90	2.753 (3)	159
N2—H2*B*⋯O5^vi^	0.89	2.07	2.829 (3)	143
N2—H2*B*⋯O7	0.89	2.24	2.677 (4)	110
C6—H6⋯O6	0.93	2.58	3.261 (4)	130

Symmetry codes: (i) 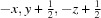; (ii) 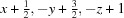; (iii) 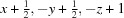; (iv) 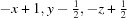; (v) 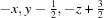; (vi) 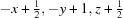.

**Table 2:** Hydrogen-bond geometry (Å, °) for (II)

*D*—H⋯*A*	*D*—H	H⋯*A*	*D*⋯*A*	*D*—H⋯*A*
O1—H1′⋯O8	0.96	1.70	2.642 (4)	169
O2—H2′⋯O6	0.82 (2)	1.83 (3)	2.647 (3)	175 (5)
O3—H3′⋯O2^i^	0.87	1.92	2.784 (3)	171
O4—H4′⋯O7^ii^	0.89 (2)	1.95 (3)	2.794 (4)	159 (4)
N1—H1*B*⋯O5^iii^	0.89	2.04	2.827 (3)	146
N1—H1*A*⋯O8^iv^	0.89	1.90	2.733 (4)	154
N2—H2*A*⋯O4^v^	0.89	2.11	2.920 (4)	150
N2—H2*B*⋯O6^vi^	0.89	1.87	2.734 (4)	163
C6—H6⋯O6	0.93	2.61	3.282 (4)	130

Symmetry codes: (i) 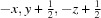; (ii) 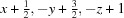; (iii) 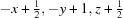; (iv) 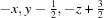; (v) 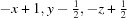; (vi) 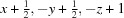.

**Table 3:** Relative percentage contributions of close contacts of PRO and OXY mol­ecules to the Hirshfeld surface of cocrystals I and II

Contacts	PRO 1	PRO 2	OXY
(I)			
H⋯H	47.9	36.6	38.6
H⋯O/O⋯H	31.4	43.6	33.3
H⋯C/C⋯H	14.6	18.8	–
(II)			
H⋯H	32.3	49.4	38.2
H⋯O/O⋯H	47.8	34.7	35.1
H⋯C/C⋯H	19.3	15.0	–

**Table 4:** Experimental details

	(I)	(II)
Crystal data		
Chemical formula	C_14_H_12_O_4_·2C_5_H_9_NO_2_·H_2_O	C_14_H_12_O_4_·2C_5_H_9_NO_2_
*M*_r_	492.51	474.50
Crystal system, space group	Orthorhombic, *P*2_1_2_1_2_1_	Orthorhombic, *P*2_1_2_1_2_1_
Temperature (K)	297	297
*a*, *b*, *c* (Å)	9.9759 (2), 10.6052 (2), 22.8535 (4)	9.8293 (2), 10.4915 (2), 22.9863 (6)
*V* (Å^3^)	2417.82 (8)	2370.44 (9)
*Z*	4	4
Radiation type	Mo *K*α	Mo *K*α
μ (mm^−1^)	0.10	0.10
Crystal size (mm)	0.33 × 0.23 × 0.11	0.46 × 0.33 × 0.19

Data collection		
Diffractometer	Bruker D8 VENTURE	Bruker D8 VENTURE
Absorption correction	Multi-scan (*SADABS*; Bruker, 2016 ▸)	Multi-scan (*SADABS*; Bruker, 2016 ▸)
*T*_min_, *T*_max_	0.716, 0.746	0.656, 0.747
No. of measured, independent and observed [*I* > 2σ(*I*)] reflections	27568, 4237, 4046	22887, 4084, 4038
*R*_int_	0.024	0.019
(sin θ/λ)_max_ (Å^−1^)	0.595	0.594

Refinement		
*R*[*F* ^2^ > 2σ(*F* ^2^)], *wR*(*F* ^2^), *S*	0.044, 0.117, 1.05	0.047, 0.134, 1.09
No. of reflections	4237	4084
No. of parameters	332	317
No. of restraints	4	2
H-atom treatment	H atoms treated by a mixture of independent and constrained refinement	H atoms treated by a mixture of independent and constrained refinement
Δρ_max_, Δρ_min_ (e Å^−3^)	0.35, −0.31	0.62, −0.22

Computer programs: *APEX2* and *SAINT* (Bruker, 2016 ▸), *SHELXT2014* (Sheldrick, 2015*a* ▸), *SHELXL2018/3* (Sheldrick, 2015*b* ▸), *Mercury* (Macrae *et al.*, 2020 ▸), *WinGX* (Farrugia, 2012 ▸) and *publCIF* (Westrip, 2010 ▸).

## supplementary crystallographic information

### 4-[(E)-2-(3,5-Dihydroxyphenyl)ethenyl]benzene-1,3-diol bis[(S)-pyrrolidin-1-ium-2-carboxylate] monohydrate (I) Crystal data

**Table:**

C_14_H_12_O_4_·2C_5_H_9_NO_2_·H_2_O	*D*_x_ = 1.353 Mg m^−^^3^
*M**_r_* = 492.51	Mo *K*α radiation, λ = 0.71073 Å
Orthorhombic, *P*2_1_2_1_2_1_	Cell parameters from 9870 reflections
*a* = 9.9759 (2) Å	θ = 3.3–27.2°
*b* = 10.6052 (2) Å	µ = 0.10 mm^−^^1^
*c* = 22.8535 (4) Å	*T* = 297 K
*V* = 2417.82 (8) Å^3^	Rod, colourless
*Z* = 4	0.33 × 0.23 × 0.11 mm
*F*(000) = 1048	

### 4-[(E)-2-(3,5-Dihydroxyphenyl)ethenyl]benzene-1,3-diol bis[(S)-pyrrolidin-1-ium-2-carboxylate] monohydrate (I) Data collection

**Table:**

Bruker D8 VENTURE diffractometer	4237 independent reflections
Radiation source: Sealed x-ray tube	4046 reflections with *I* > 2σ(*I*)
GraphiteDouble Bounce Multilayer Mirror monochromator	*R*_int_ = 0.024
Detector resolution: 7.39 pixels mm^-1^	θ_max_ = 25.0°, θ_min_ = 2.9°
φ and ω scans	*h* = −11→11
Absorption correction: multi-scan (SADABS; Bruker, 2016)	*k* = −12→12
*T*_min_ = 0.716, *T*_max_ = 0.746	*l* = −27→27
27568 measured reflections	

### 4-[(E)-2-(3,5-Dihydroxyphenyl)ethenyl]benzene-1,3-diol bis[(S)-pyrrolidin-1-ium-2-carboxylate] monohydrate (I) Refinement

**Table:**

Refinement on *F*^2^	Primary atom site location: structure-invariant direct methods
Least-squares matrix: full	Secondary atom site location: difference Fourier map
*R*[*F*^2^ > 2σ(*F*^2^)] = 0.044	Hydrogen site location: mixed
*wR*(*F*^2^) = 0.117	H atoms treated by a mixture of independent and constrained refinement
*S* = 1.05	*w* = 1/[σ^2^(*F*_o_^2^) + (0.0603*P*)^2^ + 1.0192*P*] where *P* = (*F*_o_^2^ + 2*F*_c_^2^)/3
4237 reflections	(Δ/σ)_max_ < 0.001
332 parameters	Δρ_max_ = 0.35 e Å^−^^3^
4 restraints	Δρ_min_ = −0.31 e Å^−^^3^

### 4-[(E)-2-(3,5-Dihydroxyphenyl)ethenyl]benzene-1,3-diol bis[(S)-pyrrolidin-1-ium-2-carboxylate] monohydrate (I) Special details

**Table:**

Geometry. All esds (except the esd in the dihedral angle between two l.s. planes) are estimated using the full covariance matrix. The cell esds are taken into account individually in the estimation of esds in distances, angles and torsion angles; correlations between esds in cell parameters are only used when they are defined by crystal symmetry. An approximate (isotropic) treatment of cell esds is used for estimating esds involving l.s. planes.

### 4-[(E)-2-(3,5-Dihydroxyphenyl)ethenyl]benzene-1,3-diol bis[(S)-pyrrolidin-1-ium-2-carboxylate] monohydrate (I) Fractional atomic coordinates and isotropic or equivalent isotropic displacement parameters (Å^2^)

**Table:**

	*x*	*y*	*z*	*U*_iso_*/*U*_eq_	
O1	0.1315 (3)	0.6159 (2)	0.61582 (10)	0.0526 (7)	
N1	0.7751 (3)	0.3182 (3)	0.47097 (13)	0.0448 (7)	
H1A	0.821241	0.251273	0.483057	0.054*	
H1B	0.765150	0.313168	0.432344	0.054*	
C1	0.1165 (3)	0.6442 (3)	0.55821 (13)	0.0380 (7)	
O2	0.2661 (2)	0.5415 (2)	0.42047 (10)	0.0456 (6)	
H2'	0.323 (4)	0.489 (4)	0.4405 (11)	0.068*	
N2	−0.0782 (3)	0.4926 (2)	0.79401 (11)	0.0366 (6)	
H2A	−0.032399	0.421409	0.799100	0.044*	
H2B	−0.055795	0.546139	0.822364	0.044*	
C2	0.0261 (4)	0.7335 (3)	0.53867 (14)	0.0459 (8)	
H2	−0.026401	0.778272	0.565113	0.055*	
O3	−0.2666 (3)	0.9933 (3)	0.19900 (11)	0.0567 (7)	
C3	0.0150 (4)	0.7553 (3)	0.47947 (14)	0.0484 (8)	
H3	−0.047640	0.814001	0.466565	0.058*	
O4	0.0969 (5)	0.7386 (4)	0.13945 (13)	0.0928 (13)	
H4'	0.190 (8)	0.723 (8)	0.1526 (16)	0.139*	
C4	0.0942 (3)	0.6928 (3)	0.43769 (13)	0.0369 (7)	
O5	0.5778 (3)	0.4212 (3)	0.41114 (10)	0.0520 (6)	
C5	0.1858 (3)	0.6041 (3)	0.45910 (12)	0.0323 (6)	
O6	0.4180 (2)	0.3743 (2)	0.47524 (11)	0.0489 (6)	
C6	0.1957 (3)	0.5792 (3)	0.51887 (13)	0.0354 (6)	
H6	0.255747	0.518732	0.532283	0.042*	
O7	−0.0442 (5)	0.7425 (2)	0.78719 (12)	0.0891 (13)	
C7	0.0819 (4)	0.7181 (3)	0.37485 (14)	0.0426 (7)	
H7	0.147494	0.684006	0.350660	0.051*	
O8	−0.0014 (3)	0.7457 (2)	0.69276 (10)	0.0562 (7)	
C8	−0.0133 (4)	0.7847 (4)	0.34932 (14)	0.0450 (8)	
H8	−0.078062	0.818540	0.374046	0.054*	
O9	0.3312 (5)	0.6407 (4)	0.1266 (2)	0.0987 (14)	
C9	−0.0299 (3)	0.8120 (3)	0.28670 (14)	0.0391 (7)	
C10	−0.1353 (3)	0.8902 (3)	0.27012 (14)	0.0418 (7)	
H10	−0.190263	0.925334	0.298660	0.050*	
C11	−0.1595 (3)	0.9162 (3)	0.21180 (14)	0.0426 (7)	
C12	−0.0792 (4)	0.8656 (4)	0.16910 (15)	0.0519 (9)	
H12	−0.094718	0.884207	0.129914	0.062*	
C13	0.0242 (4)	0.7874 (4)	0.18468 (15)	0.0548 (9)	
C14	0.0517 (4)	0.7593 (3)	0.24318 (15)	0.0472 (8)	
H14	0.122696	0.706643	0.253042	0.057*	
C15	0.5376 (3)	0.3770 (3)	0.45739 (13)	0.0357 (7)	
C16	0.6412 (3)	0.3223 (3)	0.50027 (14)	0.0378 (7)	
H16	0.614353	0.237957	0.513251	0.045*	
C17	0.6655 (4)	0.4086 (5)	0.55262 (16)	0.0623 (11)	
H17A	0.583267	0.450158	0.564463	0.075*	
H17B	0.700761	0.361392	0.585567	0.075*	
C18	0.7653 (6)	0.5017 (6)	0.5311 (3)	0.0917 (17)	
H18A	0.720375	0.573782	0.513840	0.110*	
H18B	0.821256	0.530953	0.563023	0.110*	
C19	0.8472 (5)	0.4362 (5)	0.4868 (3)	0.0820 (15)	
H19A	0.935075	0.416595	0.502539	0.098*	
H19B	0.858597	0.489086	0.452511	0.098*	
C20	−0.0468 (3)	0.5485 (3)	0.73547 (12)	0.0331 (6)	
H20	0.035826	0.510986	0.720132	0.040*	
C21	−0.1645 (5)	0.5115 (4)	0.69655 (18)	0.0650 (11)	
H21A	−0.210407	0.586187	0.682371	0.078*	
H21B	−0.133480	0.463214	0.663169	0.078*	
C22	−0.2580 (5)	0.4327 (5)	0.7340 (2)	0.0784 (14)	
H22A	−0.350908	0.452288	0.725335	0.094*	
H22B	−0.243179	0.343458	0.727209	0.094*	
C23	−0.2245 (4)	0.4669 (4)	0.7956 (2)	0.0656 (11)	
H23A	−0.273919	0.541110	0.807923	0.079*	
H23B	−0.244741	0.397964	0.822045	0.079*	
C24	−0.0301 (4)	0.6913 (3)	0.73993 (14)	0.0455 (8)	
H1'	0.080 (4)	0.660 (4)	0.6361 (18)	0.068*	
H3'	−0.270 (5)	1.004 (5)	0.1623 (11)	0.068*	
H9A	0.376 (4)	0.670 (4)	0.1576 (14)	0.055*	
H9B	0.419 (3)	0.644 (4)	0.1031 (16)	0.055*	

### 4-[(E)-2-(3,5-Dihydroxyphenyl)ethenyl]benzene-1,3-diol bis[(S)-pyrrolidin-1-ium-2-carboxylate] monohydrate (I) Atomic displacement parameters (Å^2^)

**Table:**

	*U*^11^	*U*^22^	*U*^33^	*U*^12^	*U*^13^	*U*^23^
O1	0.0727 (17)	0.0544 (15)	0.0305 (11)	0.0138 (13)	0.0030 (11)	0.0011 (11)
N1	0.0333 (14)	0.0529 (17)	0.0481 (16)	0.0065 (12)	0.0018 (12)	0.0036 (13)
C1	0.0474 (18)	0.0358 (16)	0.0309 (15)	−0.0028 (14)	−0.0001 (13)	−0.0031 (12)
O2	0.0475 (13)	0.0572 (14)	0.0322 (12)	0.0176 (11)	−0.0042 (10)	−0.0039 (10)
N2	0.0513 (16)	0.0274 (12)	0.0311 (13)	−0.0006 (11)	0.0041 (11)	−0.0008 (10)
C2	0.0532 (19)	0.0458 (18)	0.0388 (17)	0.0128 (16)	0.0072 (15)	−0.0022 (14)
O3	0.0526 (15)	0.0767 (18)	0.0408 (14)	0.0061 (14)	−0.0015 (11)	0.0187 (14)
C3	0.054 (2)	0.0473 (19)	0.0439 (18)	0.0172 (16)	0.0014 (16)	0.0053 (15)
O4	0.126 (3)	0.097 (3)	0.0546 (17)	0.039 (2)	0.0386 (19)	0.0031 (18)
C4	0.0368 (16)	0.0379 (16)	0.0359 (16)	0.0031 (13)	−0.0006 (13)	−0.0006 (13)
O5	0.0557 (14)	0.0676 (16)	0.0326 (11)	0.0066 (12)	0.0033 (11)	0.0128 (11)
C5	0.0310 (14)	0.0343 (15)	0.0316 (14)	−0.0030 (12)	−0.0022 (11)	−0.0052 (12)
O6	0.0313 (12)	0.0589 (15)	0.0565 (14)	0.0063 (11)	0.0013 (10)	0.0141 (12)
C6	0.0351 (15)	0.0371 (15)	0.0339 (15)	0.0014 (12)	−0.0054 (12)	0.0003 (13)
O7	0.191 (4)	0.0338 (13)	0.0430 (14)	−0.0075 (19)	0.031 (2)	−0.0107 (12)
C7	0.0484 (18)	0.0435 (18)	0.0358 (16)	0.0078 (15)	0.0034 (14)	0.0017 (14)
O8	0.099 (2)	0.0303 (11)	0.0394 (12)	−0.0038 (13)	0.0118 (13)	0.0020 (10)
C8	0.0436 (18)	0.056 (2)	0.0360 (17)	0.0085 (16)	0.0019 (14)	0.0033 (15)
O9	0.109 (3)	0.072 (2)	0.115 (3)	0.016 (2)	−0.056 (3)	−0.022 (2)
C9	0.0418 (16)	0.0414 (17)	0.0341 (15)	−0.0007 (14)	0.0015 (14)	0.0045 (13)
C10	0.0434 (17)	0.0479 (18)	0.0341 (16)	0.0007 (15)	0.0058 (13)	0.0057 (14)
C11	0.0456 (18)	0.0458 (18)	0.0363 (16)	−0.0069 (15)	−0.0001 (14)	0.0074 (14)
C12	0.072 (2)	0.052 (2)	0.0313 (16)	−0.0028 (19)	0.0020 (16)	0.0065 (15)
C13	0.074 (2)	0.0491 (19)	0.0411 (19)	0.0025 (19)	0.0206 (18)	0.0002 (16)
C14	0.054 (2)	0.0429 (18)	0.0449 (18)	0.0043 (15)	0.0050 (16)	0.0058 (15)
C15	0.0348 (16)	0.0383 (16)	0.0340 (16)	0.0034 (13)	−0.0010 (12)	0.0005 (13)
C16	0.0318 (15)	0.0457 (18)	0.0357 (15)	0.0007 (13)	0.0013 (13)	0.0089 (13)
C17	0.057 (2)	0.095 (3)	0.0356 (18)	0.000 (2)	−0.0052 (16)	−0.005 (2)
C18	0.092 (4)	0.094 (4)	0.088 (4)	−0.028 (3)	0.004 (3)	−0.033 (3)
C19	0.053 (2)	0.058 (3)	0.135 (5)	−0.012 (2)	0.018 (3)	0.000 (3)
C20	0.0461 (17)	0.0274 (13)	0.0259 (13)	0.0006 (12)	0.0021 (12)	−0.0016 (11)
C21	0.079 (3)	0.065 (3)	0.051 (2)	−0.018 (2)	−0.023 (2)	0.003 (2)
C22	0.062 (3)	0.077 (3)	0.096 (4)	−0.022 (2)	−0.018 (2)	0.004 (3)
C23	0.053 (2)	0.069 (3)	0.075 (3)	−0.005 (2)	0.015 (2)	0.007 (2)
C24	0.073 (2)	0.0290 (15)	0.0341 (16)	0.0016 (15)	0.0058 (16)	−0.0039 (13)

### 4-[(E)-2-(3,5-Dihydroxyphenyl)ethenyl]benzene-1,3-diol bis[(S)-pyrrolidin-1-ium-2-carboxylate] monohydrate (I) Geometric parameters (Å, º)

**Table:**

O1—C1	1.359 (4)	O9—H9A	0.89 (2)
O1—H1'	0.84 (2)	O9—H9B	1.03 (2)
N1—C19	1.487 (6)	C9—C10	1.392 (5)
N1—C16	1.495 (4)	C9—C14	1.401 (5)
N1—H1A	0.8900	C10—C11	1.382 (4)
N1—H1B	0.8900	C10—H10	0.9300
C1—C6	1.381 (5)	C11—C12	1.372 (5)
C1—C2	1.382 (5)	C12—C13	1.370 (6)
O2—C5	1.364 (4)	C12—H12	0.9300
O2—H2'	0.92 (5)	C13—C14	1.397 (5)
N2—C23	1.486 (5)	C14—H14	0.9300
N2—C20	1.496 (4)	C15—C16	1.538 (4)
N2—H2A	0.8900	C16—C17	1.525 (5)
N2—H2B	0.8900	C16—H16	0.9800
C2—C3	1.377 (5)	C17—C18	1.486 (7)
C2—H2	0.9300	C17—H17A	0.9700
O3—C11	1.378 (4)	C17—H17B	0.9700
O3—H3'	0.85 (2)	C18—C19	1.475 (8)
C3—C4	1.405 (5)	C18—H18A	0.9700
C3—H3	0.9300	C18—H18B	0.9700
O4—C13	1.365 (4)	C19—H19A	0.9700
O4—H4'	0.99 (8)	C19—H19B	0.9700
C4—C5	1.400 (4)	C20—C21	1.525 (5)
C4—C7	1.466 (4)	C20—C24	1.526 (4)
O5—C15	1.224 (4)	C20—H20	0.9800
C5—C6	1.395 (4)	C21—C22	1.517 (6)
O6—C15	1.262 (4)	C21—H21A	0.9700
C6—H6	0.9300	C21—H21B	0.9700
O7—C24	1.217 (4)	C22—C23	1.492 (7)
C7—C8	1.319 (5)	C22—H22A	0.9700
C7—H7	0.9300	C22—H22B	0.9700
O8—C24	1.256 (4)	C23—H23A	0.9700
C8—C9	1.469 (4)	C23—H23B	0.9700
C8—H8	0.9300		
			
C1—O1—H1'	110 (3)	C13—C14—H14	120.6
C19—N1—C16	107.4 (3)	C9—C14—H14	120.6
C19—N1—H1A	110.2	O5—C15—O6	126.7 (3)
C16—N1—H1A	110.2	O5—C15—C16	118.3 (3)
C19—N1—H1B	110.2	O6—C15—C16	114.9 (3)
C16—N1—H1B	110.2	N1—C16—C17	103.1 (3)
H1A—N1—H1B	108.5	N1—C16—C15	109.0 (2)
O1—C1—C6	117.2 (3)	C17—C16—C15	112.4 (3)
O1—C1—C2	122.5 (3)	N1—C16—H16	110.7
C6—C1—C2	120.3 (3)	C17—C16—H16	110.7
C5—O2—H2'	109.5	C15—C16—H16	110.7
C23—N2—C20	107.5 (3)	C18—C17—C16	104.2 (3)
C23—N2—H2A	110.2	C18—C17—H17A	110.9
C20—N2—H2A	110.2	C16—C17—H17A	110.9
C23—N2—H2B	110.2	C18—C17—H17B	110.9
C20—N2—H2B	110.2	C16—C17—H17B	110.9
H2A—N2—H2B	108.5	H17A—C17—H17B	108.9
C3—C2—C1	119.0 (3)	C19—C18—C17	106.6 (4)
C3—C2—H2	120.5	C19—C18—H18A	110.4
C1—C2—H2	120.5	C17—C18—H18A	110.4
C11—O3—H3'	109 (3)	C19—C18—H18B	110.4
C2—C3—C4	122.9 (3)	C17—C18—H18B	110.4
C2—C3—H3	118.6	H18A—C18—H18B	108.6
C4—C3—H3	118.6	C18—C19—N1	107.2 (4)
C13—O4—H4'	109.5	C18—C19—H19A	110.3
C5—C4—C3	116.5 (3)	N1—C19—H19A	110.3
C5—C4—C7	121.3 (3)	C18—C19—H19B	110.3
C3—C4—C7	122.2 (3)	N1—C19—H19B	110.3
O2—C5—C6	120.0 (3)	H19A—C19—H19B	108.5
O2—C5—C4	119.0 (3)	N2—C20—C21	105.0 (3)
C6—C5—C4	121.0 (3)	N2—C20—C24	110.9 (2)
C1—C6—C5	120.2 (3)	C21—C20—C24	112.2 (3)
C1—C6—H6	119.9	N2—C20—H20	109.6
C5—C6—H6	119.9	C21—C20—H20	109.6
C8—C7—C4	126.2 (3)	C24—C20—H20	109.6
C8—C7—H7	116.9	C22—C21—C20	106.6 (3)
C4—C7—H7	116.9	C22—C21—H21A	110.4
C7—C8—C9	128.1 (3)	C20—C21—H21A	110.4
C7—C8—H8	116.0	C22—C21—H21B	110.4
C9—C8—H8	116.0	C20—C21—H21B	110.4
H9A—O9—H9B	89 (3)	H21A—C21—H21B	108.6
C10—C9—C14	118.9 (3)	C23—C22—C21	105.1 (3)
C10—C9—C8	117.9 (3)	C23—C22—H22A	110.7
C14—C9—C8	123.2 (3)	C21—C22—H22A	110.7
C11—C10—C9	120.9 (3)	C23—C22—H22B	110.7
C11—C10—H10	119.6	C21—C22—H22B	110.7
C9—C10—H10	119.6	H22A—C22—H22B	108.8
C12—C11—O3	122.3 (3)	N2—C23—C22	104.0 (3)
C12—C11—C10	120.4 (3)	N2—C23—H23A	111.0
O3—C11—C10	117.3 (3)	C22—C23—H23A	111.0
C13—C12—C11	119.4 (3)	N2—C23—H23B	111.0
C13—C12—H12	120.3	C22—C23—H23B	111.0
C11—C12—H12	120.3	H23A—C23—H23B	109.0
O4—C13—C12	115.6 (3)	O7—C24—O8	125.7 (3)
O4—C13—C14	122.7 (4)	O7—C24—C20	119.3 (3)
C12—C13—C14	121.7 (3)	O8—C24—C20	115.0 (3)
C13—C14—C9	118.7 (3)		
			
O1—C1—C2—C3	−178.9 (3)	O4—C13—C14—C9	−178.9 (4)
C6—C1—C2—C3	0.9 (5)	C12—C13—C14—C9	0.6 (6)
C1—C2—C3—C4	−1.6 (6)	C10—C9—C14—C13	0.1 (5)
C2—C3—C4—C5	0.8 (5)	C8—C9—C14—C13	177.5 (4)
C2—C3—C4—C7	−179.4 (4)	C19—N1—C16—C17	−25.4 (4)
C3—C4—C5—O2	−179.4 (3)	C19—N1—C16—C15	94.1 (4)
C7—C4—C5—O2	0.8 (5)	O5—C15—C16—N1	−8.3 (4)
C3—C4—C5—C6	0.7 (5)	O6—C15—C16—N1	173.7 (3)
C7—C4—C5—C6	−179.1 (3)	O5—C15—C16—C17	105.3 (4)
O1—C1—C6—C5	−179.7 (3)	O6—C15—C16—C17	−72.7 (4)
C2—C1—C6—C5	0.5 (5)	N1—C16—C17—C18	33.9 (4)
O2—C5—C6—C1	178.7 (3)	C15—C16—C17—C18	−83.3 (4)
C4—C5—C6—C1	−1.4 (5)	C16—C17—C18—C19	−30.2 (6)
C5—C4—C7—C8	169.2 (4)	C17—C18—C19—N1	14.5 (6)
C3—C4—C7—C8	−10.6 (6)	C16—N1—C19—C18	7.3 (6)
C4—C7—C8—C9	−179.7 (3)	C23—N2—C20—C21	−19.7 (4)
C7—C8—C9—C10	−176.9 (4)	C23—N2—C20—C24	101.7 (3)
C7—C8—C9—C14	5.8 (6)	N2—C20—C21—C22	−1.4 (4)
C14—C9—C10—C11	−0.3 (5)	C24—C20—C21—C22	−121.9 (4)
C8—C9—C10—C11	−177.7 (3)	C20—C21—C22—C23	21.6 (5)
C9—C10—C11—C12	−0.3 (5)	C20—N2—C23—C22	33.4 (4)
C9—C10—C11—O3	179.3 (3)	C21—C22—C23—N2	−33.4 (5)
O3—C11—C12—C13	−178.7 (3)	N2—C20—C24—O7	−0.7 (5)
C10—C11—C12—C13	1.0 (5)	C21—C20—C24—O7	116.3 (4)
C11—C12—C13—O4	178.4 (4)	N2—C20—C24—O8	178.9 (3)
C11—C12—C13—C14	−1.1 (6)	C21—C20—C24—O8	−64.1 (5)

### 4-[(E)-2-(3,5-Dihydroxyphenyl)ethenyl]benzene-1,3-diol bis[(S)-pyrrolidin-1-ium-2-carboxylate] monohydrate (I) Hydrogen-bond geometry (Å, º)

**Table:**

*D*—H···*A*	*D*—H	H···*A*	*D*···*A*	*D*—H···*A*
O1—H1′···O8	0.84 (2)	1.78 (3)	2.597 (3)	166 (5)
O2—H2′···O6	0.92	1.74	2.647 (3)	172
O3—H3′···O2^i^	0.85 (2)	1.93 (3)	2.778 (3)	175 (5)
O4—H4′···O9	0.99	1.76	2.575 (6)	137
O9—H9*A*···O7^ii^	0.89 (2)	1.76 (3)	2.639 (5)	168 (4)
N1—H1*A*···O6^iii^	0.89	1.90	2.777 (4)	168
N1—H1*B*···O4^iv^	0.89	2.28	2.951 (4)	132
N1—H1*B*···O9^iv^	0.89	2.47	3.105 (5)	129
N2—H2*A*···O8^v^	0.89	1.90	2.753 (3)	159
N2—H2*B*···O5^vi^	0.89	2.07	2.829 (3)	143
N2—H2*B*···O7	0.89	2.24	2.677 (4)	110
C6—H6···O6	0.93	2.58	3.261 (4)	130
C12—H12···O2^i^	0.93	2.65	3.339 (5)	131

Symmetry codes: (i) −*x*, *y*+1/2, −*z*+1/2; (ii) *x*+1/2, −*y*+3/2, −*z*+1; (iii) *x*+1/2, −*y*+1/2, −*z*+1; (iv) −*x*+1, *y*−1/2, −*z*+1/2; (v) −*x*, *y*−1/2, −*z*+3/2; (vi) −*x*+1/2, −*y*+1, *z*+1/2.

### 4-[(E)-2-(3,5-Dihydroxyphenyl)ethenyl]benzene-1,3-diol bis[(S)-pyrrolidin-1-ium-2-carboxylate] (II) Crystal data

**Table:**

C_14_H_12_O_4_·2C_5_H_9_NO_2_	*D*_x_ = 1.330 Mg m^−^^3^
*M**_r_* = 474.50	Mo *K*α radiation, λ = 0.71073 Å
Orthorhombic, *P*2_1_2_1_2_1_	Cell parameters from 9885 reflections
*a* = 9.8293 (2) Å	θ = 3.0–33.7°
*b* = 10.4915 (2) Å	µ = 0.10 mm^−^^1^
*c* = 22.9863 (6) Å	*T* = 297 K
*V* = 2370.44 (9) Å^3^	Block, colourless
*Z* = 4	0.46 × 0.33 × 0.19 mm
*F*(000) = 1008	

### 4-[(E)-2-(3,5-Dihydroxyphenyl)ethenyl]benzene-1,3-diol bis[(S)-pyrrolidin-1-ium-2-carboxylate] (II) Data collection

**Table:**

Bruker D8 VENTURE diffractometer	4084 independent reflections
Radiation source: Sealed x-ray tube	4038 reflections with *I* > 2σ(*I*)
GraphiteDouble Bounce Multilayer Mirror monochromator	*R*_int_ = 0.019
Detector resolution: 7.39 pixels mm^-1^	θ_max_ = 25.0°, θ_min_ = 3.0°
φ and ω scans	*h* = −11→11
Absorption correction: multi-scan (SADABS; Bruker, 2016)	*k* = −12→12
*T*_min_ = 0.656, *T*_max_ = 0.747	*l* = −27→27
22887 measured reflections	

### 4-[(E)-2-(3,5-Dihydroxyphenyl)ethenyl]benzene-1,3-diol bis[(S)-pyrrolidin-1-ium-2-carboxylate] (II) Refinement

**Table:**

Refinement on *F*^2^	Primary atom site location: structure-invariant direct methods
Least-squares matrix: full	Secondary atom site location: difference Fourier map
*R*[*F*^2^ > 2σ(*F*^2^)] = 0.047	Hydrogen site location: mixed
*wR*(*F*^2^) = 0.134	H atoms treated by a mixture of independent and constrained refinement
*S* = 1.09	*w* = 1/[σ^2^(*F*_o_^2^) + (0.0845*P*)^2^ + 0.7969*P*] where *P* = (*F*_o_^2^ + 2*F*_c_^2^)/3
4084 reflections	(Δ/σ)_max_ < 0.001
317 parameters	Δρ_max_ = 0.62 e Å^−^^3^
2 restraints	Δρ_min_ = −0.22 e Å^−^^3^

### 4-[(E)-2-(3,5-Dihydroxyphenyl)ethenyl]benzene-1,3-diol bis[(S)-pyrrolidin-1-ium-2-carboxylate] (II) Special details

**Table:**

Geometry. All esds (except the esd in the dihedral angle between two l.s. planes) are estimated using the full covariance matrix. The cell esds are taken into account individually in the estimation of esds in distances, angles and torsion angles; correlations between esds in cell parameters are only used when they are defined by crystal symmetry. An approximate (isotropic) treatment of cell esds is used for estimating esds involving l.s. planes.

### 4-[(E)-2-(3,5-Dihydroxyphenyl)ethenyl]benzene-1,3-diol bis[(S)-pyrrolidin-1-ium-2-carboxylate] (II) Fractional atomic coordinates and isotropic or equivalent isotropic displacement parameters (Å^2^)

**Table:**

	*x*	*y*	*z*	*U*_iso_*/*U*_eq_	
O1	0.1260 (3)	0.6128 (3)	0.61986 (10)	0.0514 (7)	
H1'	0.071 (6)	0.669 (5)	0.6427 (14)	0.077*	
N1	−0.0705 (3)	0.4844 (2)	0.79572 (11)	0.0353 (6)	
H1A	−0.014333	0.419063	0.801444	0.042*	
H1B	−0.056256	0.541800	0.823585	0.042*	
C1	0.1126 (3)	0.6427 (3)	0.56307 (13)	0.0352 (7)	
O2	0.2565 (2)	0.5318 (3)	0.42496 (9)	0.0427 (6)	
N2	0.7837 (3)	0.3235 (3)	0.46631 (12)	0.0389 (6)	
H2A	0.772557	0.318253	0.427954	0.047*	
H2B	0.837980	0.260292	0.477681	0.047*	
C2	0.0265 (4)	0.7382 (4)	0.54340 (14)	0.0453 (8)	
H2	−0.024706	0.785813	0.569582	0.054*	
O3	−0.2495 (3)	0.9798 (3)	0.19378 (11)	0.0495 (6)	
H3'	−0.249 (3)	0.987 (5)	0.156 (3)	0.074*	
C3	0.0180 (4)	0.7616 (4)	0.48443 (14)	0.0455 (8)	
H3	−0.041818	0.824262	0.471595	0.055*	
O4	0.1637 (3)	0.7502 (3)	0.15416 (11)	0.0599 (8)	
C4	0.0949 (3)	0.6957 (3)	0.44306 (13)	0.0356 (7)	
O5	0.5729 (3)	0.4173 (3)	0.41022 (10)	0.0533 (7)	
C5	0.1812 (3)	0.6002 (3)	0.46420 (13)	0.0309 (6)	
O6	0.4198 (2)	0.3686 (3)	0.47823 (11)	0.0466 (6)	
C6	0.1903 (3)	0.5733 (3)	0.52325 (13)	0.0329 (6)	
H6	0.247994	0.509154	0.536261	0.039*	
O7	−0.0892 (3)	0.7406 (2)	0.78591 (10)	0.0460 (6)	
C7	0.0875 (4)	0.7241 (3)	0.38077 (13)	0.0386 (7)	
H7	0.156958	0.691646	0.357645	0.046*	
O8	−0.0296 (3)	0.7428 (2)	0.69242 (10)	0.0506 (7)	
C8	−0.0076 (3)	0.7910 (4)	0.35402 (14)	0.0398 (7)	
H8	−0.075927	0.825377	0.377158	0.048*	
C9	−0.0159 (3)	0.8164 (3)	0.29118 (14)	0.0360 (7)	
C10	−0.1234 (3)	0.8909 (3)	0.27047 (14)	0.0371 (7)	
H10	−0.185411	0.926232	0.296429	0.044*	
C11	−0.1378 (3)	0.9121 (3)	0.21109 (14)	0.0366 (7)	
C12	−0.0429 (4)	0.8644 (3)	0.17235 (13)	0.0411 (8)	
H12	−0.051837	0.880042	0.132705	0.049*	
C13	0.0660 (4)	0.7930 (3)	0.19307 (14)	0.0419 (7)	
C14	0.0793 (4)	0.7665 (3)	0.25177 (14)	0.0399 (7)	
H14	0.150671	0.716115	0.265004	0.048*	
C15	0.5388 (3)	0.3722 (3)	0.45688 (13)	0.0329 (6)	
C16	0.6496 (3)	0.3150 (3)	0.49651 (14)	0.0378 (7)	
H16	0.628213	0.226093	0.505799	0.045*	
C17	0.6714 (5)	0.3919 (6)	0.55258 (16)	0.0633 (12)	
H17A	0.588047	0.434238	0.564351	0.076*	
H17B	0.701960	0.337248	0.583990	0.076*	
C18	0.7786 (8)	0.4871 (8)	0.5367 (3)	0.108 (3)	
H18A	0.737801	0.570706	0.532318	0.130*	
H18B	0.845990	0.491800	0.567471	0.130*	
C19	0.8440 (5)	0.4497 (5)	0.4825 (2)	0.0664 (12)	
H19A	0.941535	0.442251	0.487748	0.080*	
H19B	0.826332	0.512272	0.452326	0.080*	
C20	−0.0456 (3)	0.5426 (3)	0.73720 (12)	0.0326 (6)	
H20	0.044694	0.517884	0.723075	0.039*	
C21	−0.1553 (5)	0.4849 (4)	0.69788 (18)	0.0583 (10)	
H21A	−0.208778	0.551754	0.679827	0.070*	
H21B	−0.113911	0.433781	0.667490	0.070*	
C22	−0.2429 (6)	0.4041 (6)	0.7356 (2)	0.0830 (17)	
H22A	−0.338064	0.418173	0.726516	0.100*	
H22B	−0.222303	0.314678	0.729493	0.100*	
C23	−0.2149 (4)	0.4400 (4)	0.7970 (2)	0.0583 (10)	
H23A	−0.225509	0.367342	0.822688	0.070*	
H23B	−0.275284	0.507642	0.809722	0.070*	
C24	−0.0559 (3)	0.6875 (3)	0.74016 (13)	0.0350 (7)	
H2'	0.311 (4)	0.484 (4)	0.4408 (18)	0.052*	
H4'	0.234 (3)	0.736 (5)	0.1775 (16)	0.052*	

### 4-[(E)-2-(3,5-Dihydroxyphenyl)ethenyl]benzene-1,3-diol bis[(S)-pyrrolidin-1-ium-2-carboxylate] (II) Atomic displacement parameters (Å^2^)

**Table:**

	*U*^11^	*U*^22^	*U*^33^	*U*^12^	*U*^13^	*U*^23^
O1	0.0627 (15)	0.0674 (16)	0.0241 (11)	0.0127 (14)	0.0039 (11)	0.0043 (11)
N1	0.0470 (15)	0.0298 (11)	0.0291 (12)	0.0001 (11)	0.0021 (11)	−0.0007 (10)
C1	0.0366 (15)	0.0449 (17)	0.0242 (14)	−0.0039 (13)	0.0001 (12)	−0.0004 (12)
O2	0.0439 (13)	0.0569 (15)	0.0272 (11)	0.0194 (11)	−0.0044 (10)	−0.0057 (10)
N2	0.0297 (13)	0.0499 (15)	0.0372 (14)	0.0040 (12)	0.0003 (11)	0.0029 (12)
C2	0.0494 (18)	0.0534 (19)	0.0330 (16)	0.0142 (16)	0.0088 (14)	−0.0023 (15)
O3	0.0459 (13)	0.0668 (16)	0.0357 (12)	0.0036 (12)	−0.0028 (10)	0.0172 (12)
C3	0.0517 (19)	0.0494 (18)	0.0355 (17)	0.0193 (17)	0.0019 (14)	0.0031 (15)
O4	0.0617 (17)	0.082 (2)	0.0356 (13)	0.0123 (16)	0.0113 (12)	0.0022 (14)
C4	0.0369 (15)	0.0426 (16)	0.0274 (14)	0.0044 (13)	−0.0007 (12)	0.0021 (12)
O5	0.0467 (13)	0.0819 (19)	0.0311 (11)	0.0097 (13)	0.0015 (10)	0.0159 (12)
C5	0.0261 (13)	0.0385 (15)	0.0281 (14)	−0.0001 (12)	0.0009 (11)	−0.0053 (12)
O6	0.0301 (11)	0.0578 (14)	0.0517 (14)	0.0071 (11)	0.0051 (10)	0.0140 (11)
C6	0.0318 (14)	0.0375 (15)	0.0294 (15)	0.0028 (12)	−0.0035 (12)	0.0009 (12)
O7	0.0643 (16)	0.0357 (11)	0.0382 (12)	−0.0033 (11)	0.0102 (11)	−0.0075 (10)
C7	0.0435 (16)	0.0425 (16)	0.0297 (14)	0.0083 (14)	0.0031 (13)	0.0020 (13)
O8	0.0834 (19)	0.0336 (11)	0.0347 (12)	−0.0046 (12)	0.0123 (12)	0.0041 (10)
C8	0.0374 (16)	0.0523 (18)	0.0295 (15)	0.0059 (14)	0.0017 (12)	0.0023 (14)
C9	0.0381 (16)	0.0403 (15)	0.0297 (15)	−0.0020 (13)	0.0008 (13)	0.0034 (13)
C10	0.0366 (16)	0.0436 (16)	0.0310 (15)	0.0000 (14)	0.0020 (12)	0.0030 (13)
C11	0.0376 (16)	0.0406 (16)	0.0315 (15)	−0.0057 (13)	−0.0039 (13)	0.0090 (13)
C12	0.0518 (19)	0.0478 (17)	0.0236 (14)	−0.0086 (16)	−0.0010 (13)	0.0062 (13)
C13	0.0485 (19)	0.0450 (17)	0.0324 (16)	−0.0022 (15)	0.0075 (14)	−0.0001 (13)
C14	0.0417 (16)	0.0448 (17)	0.0333 (15)	0.0014 (14)	0.0018 (13)	0.0040 (13)
C15	0.0305 (15)	0.0389 (15)	0.0294 (15)	0.0020 (13)	−0.0008 (12)	0.0029 (12)
C16	0.0323 (15)	0.0458 (17)	0.0354 (17)	0.0018 (13)	0.0001 (13)	0.0105 (14)
C17	0.058 (2)	0.103 (3)	0.0294 (17)	0.004 (2)	−0.0044 (16)	−0.002 (2)
C18	0.111 (5)	0.121 (5)	0.093 (4)	−0.052 (4)	0.023 (4)	−0.062 (4)
C19	0.054 (2)	0.063 (3)	0.083 (3)	−0.018 (2)	0.005 (2)	−0.003 (2)
C20	0.0404 (15)	0.0294 (13)	0.0280 (14)	−0.0022 (12)	0.0035 (12)	−0.0017 (11)
C21	0.078 (3)	0.053 (2)	0.043 (2)	−0.014 (2)	−0.0174 (19)	−0.0079 (17)
C22	0.073 (3)	0.089 (3)	0.088 (4)	−0.044 (3)	−0.027 (3)	0.008 (3)
C23	0.049 (2)	0.057 (2)	0.069 (3)	−0.0053 (18)	0.0188 (19)	0.012 (2)
C24	0.0420 (16)	0.0321 (14)	0.0309 (14)	−0.0060 (13)	0.0039 (13)	−0.0023 (12)

### 4-[(E)-2-(3,5-Dihydroxyphenyl)ethenyl]benzene-1,3-diol bis[(S)-pyrrolidin-1-ium-2-carboxylate] (II) Geometric parameters (Å, º)

**Table:**

O1—C1	1.349 (4)	C8—H8	0.9300
O1—H1'	0.96 (6)	C9—C10	1.398 (5)
N1—C23	1.495 (5)	C9—C14	1.403 (5)
N1—C20	1.497 (4)	C10—C11	1.390 (4)
N1—H1A	0.8900	C10—H10	0.9300
N1—H1B	0.8900	C11—C12	1.383 (5)
C1—C2	1.387 (5)	C12—C13	1.391 (5)
C1—C6	1.397 (5)	C12—H12	0.9300
O2—C5	1.369 (4)	C13—C14	1.384 (4)
O2—H2'	0.82 (2)	C14—H14	0.9300
N2—C16	1.492 (4)	C15—C16	1.541 (4)
N2—C19	1.498 (5)	C16—C17	1.535 (5)
N2—H2A	0.8900	C16—H16	0.9800
N2—H2B	0.8900	C17—C18	1.497 (8)
C2—C3	1.380 (5)	C17—H17A	0.9700
C2—H2	0.9300	C17—H17B	0.9700
O3—C11	1.367 (4)	C18—C19	1.456 (8)
O3—H3'	0.87 (6)	C18—H18A	0.9700
C3—C4	1.398 (5)	C18—H18B	0.9700
C3—H3	0.9300	C19—H19A	0.9700
O4—C13	1.387 (4)	C19—H19B	0.9700
O4—H4'	0.89 (2)	C20—C24	1.526 (4)
C4—C5	1.400 (4)	C20—C21	1.531 (5)
C4—C7	1.464 (4)	C20—H20	0.9800
O5—C15	1.219 (4)	C21—C22	1.488 (7)
C5—C6	1.389 (4)	C21—H21A	0.9700
O6—C15	1.269 (4)	C21—H21B	0.9700
C6—H6	0.9300	C22—C23	1.485 (7)
O7—C24	1.234 (4)	C22—H22A	0.9700
C7—C8	1.320 (5)	C22—H22B	0.9700
C7—H7	0.9300	C23—H23A	0.9700
O8—C24	1.268 (4)	C23—H23B	0.9700
C8—C9	1.471 (4)		
			
C1—O1—H1'	109.5	C13—C14—H14	120.3
C23—N1—C20	107.4 (3)	C9—C14—H14	120.3
C23—N1—H1A	110.2	O5—C15—O6	127.2 (3)
C20—N1—H1A	110.2	O5—C15—C16	118.5 (3)
C23—N1—H1B	110.2	O6—C15—C16	114.3 (3)
C20—N1—H1B	110.2	N2—C16—C17	103.7 (3)
H1A—N1—H1B	108.5	N2—C16—C15	109.0 (3)
O1—C1—C2	122.9 (3)	C17—C16—C15	113.0 (3)
O1—C1—C6	117.4 (3)	N2—C16—H16	110.3
C2—C1—C6	119.8 (3)	C17—C16—H16	110.3
C5—O2—H2'	112 (3)	C15—C16—H16	110.3
C16—N2—C19	106.6 (3)	C18—C17—C16	104.1 (3)
C16—N2—H2A	110.4	C18—C17—H17A	110.9
C19—N2—H2A	110.4	C16—C17—H17A	110.9
C16—N2—H2B	110.4	C18—C17—H17B	110.9
C19—N2—H2B	110.4	C16—C17—H17B	110.9
H2A—N2—H2B	108.6	H17A—C17—H17B	109.0
C3—C2—C1	119.0 (3)	C19—C18—C17	109.8 (4)
C3—C2—H2	120.5	C19—C18—H18A	109.7
C1—C2—H2	120.5	C17—C18—H18A	109.7
C11—O3—H3'	109.5	C19—C18—H18B	109.7
C2—C3—C4	123.2 (3)	C17—C18—H18B	109.7
C2—C3—H3	118.4	H18A—C18—H18B	108.2
C4—C3—H3	118.4	C18—C19—N2	106.0 (4)
C13—O4—H4'	102 (3)	C18—C19—H19A	110.5
C3—C4—C5	116.5 (3)	N2—C19—H19A	110.5
C3—C4—C7	122.5 (3)	C18—C19—H19B	110.5
C5—C4—C7	121.0 (3)	N2—C19—H19B	110.5
O2—C5—C6	120.2 (3)	H19A—C19—H19B	108.7
O2—C5—C4	118.3 (3)	N1—C20—C24	110.8 (2)
C6—C5—C4	121.5 (3)	N1—C20—C21	104.7 (3)
C5—C6—C1	120.0 (3)	C24—C20—C21	111.9 (3)
C5—C6—H6	120.0	N1—C20—H20	109.7
C1—C6—H6	120.0	C24—C20—H20	109.7
C8—C7—C4	126.8 (3)	C21—C20—H20	109.7
C8—C7—H7	116.6	C22—C21—C20	106.8 (3)
C4—C7—H7	116.6	C22—C21—H21A	110.4
C7—C8—C9	126.4 (3)	C20—C21—H21A	110.4
C7—C8—H8	116.8	C22—C21—H21B	110.4
C9—C8—H8	116.8	C20—C21—H21B	110.4
C10—C9—C14	119.5 (3)	H21A—C21—H21B	108.6
C10—C9—C8	118.5 (3)	C23—C22—C21	107.6 (3)
C14—C9—C8	122.0 (3)	C23—C22—H22A	110.2
C11—C10—C9	120.0 (3)	C21—C22—H22A	110.2
C11—C10—H10	120.0	C23—C22—H22B	110.2
C9—C10—H10	120.0	C21—C22—H22B	110.2
O3—C11—C12	122.8 (3)	H22A—C22—H22B	108.5
O3—C11—C10	116.8 (3)	C22—C23—N1	103.7 (3)
C12—C11—C10	120.4 (3)	C22—C23—H23A	111.0
C11—C12—C13	119.6 (3)	N1—C23—H23A	111.0
C11—C12—H12	120.2	C22—C23—H23B	111.0
C13—C12—H12	120.2	N1—C23—H23B	111.0
C14—C13—O4	119.9 (3)	H23A—C23—H23B	109.0
C14—C13—C12	121.0 (3)	O7—C24—O8	125.8 (3)
O4—C13—C12	119.1 (3)	O7—C24—C20	120.3 (3)
C13—C14—C9	119.5 (3)	O8—C24—C20	113.8 (3)
			
O1—C1—C2—C3	−179.3 (4)	O4—C13—C14—C9	176.4 (3)
C6—C1—C2—C3	0.9 (5)	C12—C13—C14—C9	−2.3 (5)
C1—C2—C3—C4	−1.7 (6)	C10—C9—C14—C13	0.7 (5)
C2—C3—C4—C5	1.4 (6)	C8—C9—C14—C13	179.7 (3)
C2—C3—C4—C7	−178.2 (4)	C19—N2—C16—C17	−31.3 (4)
C3—C4—C5—O2	178.9 (3)	C19—N2—C16—C15	89.3 (3)
C7—C4—C5—O2	−1.5 (5)	O5—C15—C16—N2	−2.2 (4)
C3—C4—C5—C6	−0.4 (5)	O6—C15—C16—N2	179.3 (3)
C7—C4—C5—C6	179.3 (3)	O5—C15—C16—C17	112.5 (4)
O2—C5—C6—C1	−179.6 (3)	O6—C15—C16—C17	−66.0 (4)
C4—C5—C6—C1	−0.3 (5)	N2—C16—C17—C18	27.9 (5)
O1—C1—C6—C5	−179.7 (3)	C15—C16—C17—C18	−89.9 (5)
C2—C1—C6—C5	0.1 (5)	C16—C17—C18—C19	−14.9 (8)
C3—C4—C7—C8	−15.6 (6)	C17—C18—C19—N2	−4.2 (8)
C5—C4—C7—C8	164.8 (3)	C16—N2—C19—C18	22.5 (6)
C4—C7—C8—C9	−178.4 (3)	C23—N1—C20—C24	99.1 (3)
C7—C8—C9—C10	−179.2 (4)	C23—N1—C20—C21	−21.8 (4)
C7—C8—C9—C14	1.8 (6)	N1—C20—C21—C22	3.7 (5)
C14—C9—C10—C11	1.8 (5)	C24—C20—C21—C22	−116.5 (4)
C8—C9—C10—C11	−177.2 (3)	C20—C21—C22—C23	15.9 (6)
C9—C10—C11—O3	176.4 (3)	C21—C22—C23—N1	−29.0 (5)
C9—C10—C11—C12	−2.8 (5)	C20—N1—C23—C22	31.5 (4)
O3—C11—C12—C13	−178.0 (3)	N1—C20—C24—O7	−4.1 (4)
C10—C11—C12—C13	1.1 (5)	C21—C20—C24—O7	112.4 (4)
C11—C12—C13—C14	1.4 (5)	N1—C20—C24—O8	176.9 (3)
C11—C12—C13—O4	−177.3 (3)	C21—C20—C24—O8	−66.5 (4)

### 4-[(E)-2-(3,5-Dihydroxyphenyl)ethenyl]benzene-1,3-diol bis[(S)-pyrrolidin-1-ium-2-carboxylate] (II) Hydrogen-bond geometry (Å, º)

**Table:**

*D*—H···*A*	*D*—H	H···*A*	*D*···*A*	*D*—H···*A*
O1—H1′···O8	0.96	1.70	2.642 (4)	169
O2—H2′···O6	0.82 (2)	1.83 (3)	2.647 (3)	175 (5)
O3—H3′···O2^i^	0.87	1.92	2.784 (3)	171
O4—H4′···O7^ii^	0.89 (2)	1.95 (3)	2.794 (4)	159 (4)
N1—H1*B*···O5^iii^	0.89	2.04	2.827 (3)	146
N1—H1*A*···O8^iv^	0.89	1.90	2.733 (4)	154
N2—H2*A*···O4^v^	0.89	2.11	2.920 (4)	150
N2—H2*B*···O6^vi^	0.89	1.87	2.734 (4)	163
C6—H6···O6	0.93	2.61	3.282 (4)	130

Symmetry codes: (i) −*x*, *y*+1/2, −*z*+1/2; (ii) *x*+1/2, −*y*+3/2, −*z*+1; (iii) −*x*+1/2, −*y*+1, *z*+1/2; (iv) −*x*, *y*−1/2, −*z*+3/2; (v) −*x*+1, *y*−1/2, −*z*+1/2; (vi) *x*+1/2, −*y*+1/2, −*z*+1.
